# Long-term follow-up of height and weight after propranolol treatment for infantile hemangiomas

**DOI:** 10.1016/j.jdin.2026.05.009

**Published:** 2026-05-18

**Authors:** Jianning Sun, Ruixue Zhao, Ran Huo, Jianhai Bi, Zhiyu Li, Xueqing Li

**Affiliations:** a Department of Plastic and Aesthetic Surgery, Shandong Provincial Hospital, Cheeloo College of Medicine, Shandong University, Jinan, China; b Department of Plastic and Aesthetic Surgery, Shandong Provincial Hospital Affiliated to Shandong First Medical University, Jinan, China; c Medical Science and Technology Innovation Center, Shandong First Medical University & Shandong Academy of Medical Sciences, Jinan, China; d Department of Plastic Surgery, The Third Affiliated Hospital of Guangzhou Medical University, Guangzhou, China

**Keywords:** adverse event, height, infantile hemangionmas, physical development, propranolol, weight, epidemiology

*To the Editor:* Oral propranolol is recognized as the primary treatment for infantile hemangiomas (IHs).^1^ Despite the generally favorable safety profile of propranolol, there also have been reports of adverse events.^2^ In a previous prospective study conducted by our team, we included 439 infants and young children who were administered oral propranolol as part of the observational cohort.^3^ Our findings indicated that initiating treatment after the age of 3 months may mitigate the risk of adverse reactions related to the central nervous system. Moreover, the administration of propranolol did not appear to influence the children's height and weight by the age of 2 years. This study seeks to extend the longitudinal monitoring of the children by assessing their height and weight 5-7 years post-medication.

Between September 2016 and July 2018, a total of 439 patients were administered with outpatient propranolol for IHs at the plastic surgery, and all had completed their initial follow-up assessments. At the conclusion of the follow-up period, patients who had undergone systemic corticosteroid therapy for a duration exceeding 2 weeks were excluded from the study. Every-12-hour propranolol was started from 0.5 mg/kg/day to 2 mg/kg/day on Day 14.^3^ The benchmarks for physical development are derived from data provided by the National Disease Control and Prevention Administration of the People's Republic of China.^4^^,^^5^ Data were analyzed using the SPSS 27.0 software (IBM). T test was used to determine statistical differences between 2 quantitative data groups. A univariate analysis (chi-squared or Fisher's exact test) was performed with the incidence of stunting, wasting, and overweight as the dependent variables. Set α to 0.05.

In a previous study involving 439 children, only 190 participants (43.28%) were subsequently followed up and included in this investigation (Table I). Among these participants, 32 individuals (16.8%) experienced adverse reactions during medication, including 22 cases in the digestive system and 10 cases in the nervous system. Notably, all adverse reactions have since resolved.

**Table I:** Baseline characteristics at time of inclusion

Characteristic	Group	*n* = 190 (%)
Age	≤3 mo	92 (48.4)
	>3 mo	98 (51.6)
Gender	Female	135 (71.1)
	Male	55 (28.9)
Medical history	None	165 (86.9)
	Preterm birth	20 (10.5)
	Others∗	4 (2.1)
	Heart diseases	1 (0.5)
Location	Trunk/extremities	66 (34.7)
	Head/neck	105 (55.3)
	Perineum/female breast	19 (10)
AEs	No	158 (83.2)
	Yes	32 (16.8)
GI symptom	No	170 (89.5)
	Yes	20 (10.5)
CNS symptom	No	180 (94.7)
	Yes	10 (5.3)
Medication courses	≤1 y	126 (66.3)
	>1 y	64 (33.7)

*AEs*, Adverse effects; *CNS*, central nervous system; *GI*, gastrointestinal.

∗ The 4 others are as follows: (1) intrauterine distress and neonatal hypoxia, (2) allergies to breast milk, cow's milk, and protein, (3) a red blood cell count of 3.03 × 10^12^/L, hemoglobin level of 92 g/L, and disrupted nocturnal sleep characterized by waking 7-8 times per night, and (4) a hemoglobin level of 98 g/L.

At the time of follow-up, arranging from 6 to 10 years old, 56 participants were assessed 7 years, 106 participants were assessed 6 years, and 28 participants were assessed 5 years post-treatment. Unexpectedly, the oral administration of propranolol did not yield a statistically significant impact on the height and weight of children under the age of 7. However, a statistically significant difference was observed in the weight of children over the age of 8 (Table II). Univariate analysis revealed insufficient evidence to establish statistically significant association between stunting, wasting, and overweight with variables such as varying starting ages, genders, medical histories, locations of IHs, medication courses, additional therapies, and short-term adverse reactions (*P* > 0.05) (Supplementary Material, available via Mendeley at https://data.mendeley.com/datasets/6j8zv3dg5k/1).

**Table II:** A comparative analysis of height, weight, and the growth and development standards for Chinese children

	Age (y)	6	7	8	9	10
Height ($\bar{x}$ ± s, cm)						
Male	Number	5	19	31	0	0
	Object	123.00 ± 10.93	126.58 ± 7.85	128.61 ± 6.83		
	Nation	121.71 ± 5.37	126.87 ± 5.74	132.40 ± 5.91	137.76 ± 6.43	137.76 ± 6.43
	*P*	.805	.873	.004∗		
Female	Number	7	48	75	4	1
	Object	121.86 ± 6.84	123.69 ± 7.98	128.95 ± 6.93	135.00 ± 5.77	120
	Nation	120.20 ± 5.16	125.46 ± 5.63	131.29 ± 6.08	137.34 ± 6.95	143.92 ± 7.36
	*P*	.545	.130	.005∗	.477	
Weight ($\bar{x}$ ± s, kg)						
Male	Number	5	19	31	0	0
	Object	22.80 ± 7.50	27.55 ± 7.75	27.92 ± 5.68		
	Nation	24.20 ± 4.97	26.94 ± 5.89	30.26 ± 6.91	34.20 ± 8.34	38.59 ± 9.81
	*P*	.698	.734	.029∗		
Female	Number	7	48	75	4	1
	Object	22.93 ± 3.30	24.92 ± 4.64	26.30 ± 5.51	30.75 ± 4.92	29
	Nation	22.65 ± 4.01	25.09 ± 4.83	28.23 ± 5.89	32.13 ± 7.54	36.85 ± 8.85
	*P*	.831	.797	.003∗	.614	

∗ *P* < .05.

This study examined the height and body weight of 190 children who were administered propranolol at a dosage of 2 mg/kg/d for the treatment of IHs over a period of 5 to 7 years. The findings of the study suggest that the oral administration of propranolol does not significantly affect the height and weight of children under the age of 7. With the low statistical power (due to the small number of outcome events), the analysis did not detect any statistically significant influence of treatment timing, duration, adverse events, or other covariates. Compared with national growth benchmarks, children in the 8-year-old subgroup had lower mean height and weight; however, the absence of an untreated control group, small subgroup sizes, and substantial loss to follow-up preclude causal attribution to propranolol. Elevated dropout rates have the potential to introduce selection bias. Further long-term follow-up and comprehensive research are necessary to investigate this potential effect.

## Conflicts of interest

None disclosed.
